# Assessment of Selected Clock Proteins (CLOCK and CRY1) and Their Relationship with Biochemical, Anthropometric, and Lifestyle Parameters in Hypertensive Patients

**DOI:** 10.3390/biom11040517

**Published:** 2021-03-30

**Authors:** Aniceta Ada Mikulska, Teresa Grzelak, Marta Pelczyńska, Paweł Bogdański, Krystyna Czyżewska

**Affiliations:** 1 Chair and Department of Physical Pharmacy and Pharmacokinetics, Poznan University of Medical Sciences, 6 Święcickiego Street, 60-781 Poznań, Poland; amikulska@ump.edu.pl; 2 Chair and Department of Physiology, Poznan University of Medical Sciences, 6 Święcickiego Street, 60-781 Poznań, Poland; 3 Chair and Department of Treatment of Obesity, Metabolic Disorders and Clinical Dietetics, Poznan University of Medical Sciences, 84 Szamarzewskiego Street, 60-569 Poznań, Poland; mpelczynska@ump.edu.pl (M.P.); pbogdanski@ump.edu.pl (P.B.); 4 Department of Nursing, Stanislaw Staszic State University of Applied Sciences in Pila, 10 Podchorążych Street, 64-920 Piła, Poland; czyzew@ump.edu.pl

**Keywords:** circadian clock, cryptochrome 1, circadian locomotor output cycles kaput, hypertension, total antioxidant status, diet, biological clocks

## Abstract

Background: Circadian rhythms misalignment is associated with hypertension. The aim of the study was to evaluate the concentration of selected clock proteins—cryptochrome 1 (CRY1) and circadian locomotor output cycles kaput (CLOCK) to determine their relationships with biochemical and anthropometric parameters and lifestyle elements (diet, physical activity, and quality of sleep) in hypertensive patients. Methods: In 31 females with hypertension (HT) and 55 non-hypertensive women (NHT) the CRY1 and CLOCK concentrations, total antioxidant status (TAS), lipid profile, and glycemia were analyzed. Blood pressure and anthropometric measurements, nutritional, exercise, and sleep analyses were performed. Results: In the HT group, the CRY1 level was 37.38% lower than in the NHT group. No differences were noted in CLOCK concentration between groups. BMI, FBG, and TG were higher in the HT group compared to the NHT group, while TC, LDL, and HDL levels were similar. The study showed no relationship between CRY1 or CLOCK concentrations and glucose or lipids profile, amount of physical activity, or sleep quality, although CRY1 was associated with some anthropometric indicators. In the HT group, increased CLOCK and CRY1 values were associated with a high TAS level. Conclusions: The serum level of CRY1 could be considered in a detailed diagnostic of hypertension risk in populations with abnormal anthropometric indices.

## 1. Introduction

The circadian clock is an endogenous mechanism that generates and synchronizes the course of many physiological processes over time. It acts as a primary regulator of adapting internal mechanisms to external environmental factors. The circadian clock generates endogenous 24 h rhythms. It controls diurnal variations in behavioral and physiological functions such as sleep-wake regulation, blood pressure (BP), lipid synthesis and metabolism, and hormone secretion [1,2,3,4,5]. Circadian rhythms are controlled by the circadian clock that consists of a central clock and peripheral clocks. The central clock is located in the suprachiasmatic nucleus (SCN) within the hypothalamus and synchronized by light/dark signals. SCN receives ganglionic input from retinal photosensitive cells. Peripheral oscillators are found in almost all tissues, including the liver, intestine, and adipose tissue [3,5,6,7,8]. Peripheral clocks predominate in local physiological cycles, including heart rate, glucose, and lipid homeostasis, secretion of some hormones and digestive juices, normalization of body temperature, and functioning of the digestive system. These oscillators are synchronized in response to diet and light cues and communicating through hormonal and neuronal signals. Desynchronization of the circadian system, such as shift work, is associated with increased risk for many adverse health effects, including hypertension [8,9,10]. For example, studies have shown that SCN ablation resulted in the disappearance of daily rhythmic blood pressure variations [11].

The molecular mechanism regulating circadian rhythms is based on the transcription-translation feedback loop (TTFL), which produces oscillations in the expression of circadian clock genes and protein activity, controlling quantitative changes in their subordinate genes CCGs (clock-controlled genes), which ultimately regulate the course of physiological processes in most body cells [5,12,13]. At the feedback center, there are two active transcription factors—CLOCK (circadian locomotor output cycles kaput) and BMAL1 (Brain-Muscle-Arnt-like 1)—which form a heterodimeric complex and bind to E-box sequences mediating transcription of Period (Per1, Per2, and Per3) and Cryptochrome (Cry1 and Cry2) genes. This leads to a gradual accumulation of protein products of these genes throughout the day. The negative feedback loop consists of the periods and cryptochromes proteins that inhibit CLOCK- and BMAL1-mediated transcription [5,6,12,14,15,16]. In addition to the primary loop, there are many interactions between biological clock genes, their proteins, and key biochemical pathways regulating intracellular metabolism [15,17]. The proteins encoded by clock genes perform important functions in various metabolic processes, regulating biological functions as part of circadian monitoring [18].

Recent publications have present the role of the circadian clock genes in the regulation of processes in the heart, kidney, blood vessels, and metabolic organs, which are essential for blood pressure regulation. Convincing evidence from many animal studies shows a relevant role for the circadian clock in the regulation of blood pressure [19,20].

Circadian clock proteins play an essential role in regulating and coordinating metabolism because most clock deficient animal models present metabolic abnormalities. Cryptochromes appear relevant to the pathogenesis of metabolic syndrome (MetS) because they participate in glucocorticoid regulation of gluconeogenesis and steroidogenesis. CRY proteins inhibit fasting-induced gluconeogenesis in the liver by influencing glucocorticoid signaling: while CRY1 and CRY2 directly interact with the glucocorticoid receptor [21]. Additionally, circadian disruption may lead to changes in the secretion of hormones such as aldosterone, renin, angiotensin II, and glucocorticoids, which are partly responsible for the daily regulation rhythm of blood pressure and inflammation [8,22].

Blood pressure exhibits a daily rhythm that has clinical value. In healthy people, blood pressure drops during nocturnal sleep compared to diurnal wakefulness. The nocturnal reduction in blood pressure is called “dipping”. Lowering blood pressure at night from 10% to 20% of daily blood pressure levels is indicative of the dipping response [20,23,24,25,26,27]. “Non-dippers” are individuals who have nocturnal blood pressure that decrease by less than 10% compared to diurnal blood pressure [20,25,26,27]. Studies showed that “non-dippers” have an increased risk of metabolic disorders, cardiovascular diseases (CVD), the progression of chronic kidney disease, mortality, and complications for individuals already diagnosed with hypertension [20,24,27,28,29].

Variation in BP during a day depends on internal and exogenous factors. Internal factors include autonomic nervous activity and humoral factors (cortisol, renin, vasoactive intestinal peptide, and aldosterone). External factors contain physical activity, diet, emotional state, and sleep/awake states [22,30]. Peripheral clock regulators of blood pressure are kidneys, the brain and nervous system, and of course—the vasculature and heart [23].

Studies have concluded an association between sleep deprivation, excessive exposure to artificial light at night, shift work schedule, or altered mealtime with metabolic imbalances that can change the circadian system and lead to dysregulation of metabolic homeostasis [7,8,31,32,33,34]. Several studies have reported that the desynchronization of the circadian pattern of BP is associated with increased risk for cardiovascular disease, but the mechanism of this dysregulation is still unknown [23,31,35,36]. Moreover, circadian misalignment increases 24 h blood pressure and decreases parasympathetic activity, and this chronic condition leads to the development of cardiovascular diseases [31,36]. A cross-sectional study showed that disruption of the BP circadian rhythm is also associated with metabolic syndrome in the male population [37]. Other studies confirmed that the circadian misalignment in healthy adults and shift workers could cause a significant increase in blood pressure and inflammatory markers and lead to CVD [31,36], and maintaining normal blood pressure is associated with decreased risk of cardiovascular events and death [38].

Most of the mechanisms underlying the role of CRY1 and CLOCK in a human organism have not been explained. To the best of our knowledge, yet there are no published results on the associations between circadian clock proteins serum concentrations and metabolic disorders. The aim of the study was to evaluate the serum concentrations of CRY1 and CLOCK and to determine their associations with biochemical and anthropometric parameters and individual lifestyle elements (diet, physical activity, and quality of sleep) in hypertensive patients.

## 2. Materials and Methods

### 2.1. Study Population

We analyzed 86 Caucasian women. Thirty-one women (age range 24–66 years) were included in the hypertensive patient group (HT, study group). Patients were recruited at the Department of Internal Medicine, Metabolic Disorders and Hypertension (Poznan University of Medical Sciences)—i.e., women with an early diagnosis of hypertension and taking antihypertensive medication. Additionally, 55 non-hypertensive female subjects (age range 25–63 years) were recruited as controls (NHT). Exclusion criteria in the study group included secondary obesity or secondary hypertension, a positive history of cancer in the last five years, chronic heart or liver failure, autoimmune diseases, hepatic, renal, adrenal, or thyroid disorders, and pregnancy or breastfeeding. The control group comprised healthy females of a similar age as those in the study group. Female who had poor health (mental or somatic), according to physical examination and laboratory analyses, following an interview or were pregnant or breastfeeding, were excluded from the control group. All participants had a similar circadian rhythm (shift workers were not included in the study).

The Local Bioethics Committee has given its permission to conduct the research project (regulation no. 729/17 and 326/18). Participation in the analysis was voluntary. Each participant provided written informed consent after having been informed about the project’s purpose and course. The study was carried out in accordance with the Declaration of Helsinki [39].

### 2.2. Study Design

#### 2.2.1. Blood Pressure Measurement

Blood pressure was measured manually three times in the morning with the patient sitting using a sphygmomanometer and an appropriate-sized cuff after 10 min of rest while patients were seated, according to standard guidelines of the European Society of Cardiology (ESC) and the European Society of Hypertension (ESH) [24]. In accordance with the ESC/ESH guidelines, hypertension was defined as office systolic blood pressure (SBP) values at least 140 mmHg and/or diastolic blood pressure (DBP) values at least 90 mmHg [24]. Most of the other definitions are also based on these criteria [40].

#### 2.2.2. Anthropometric Parameters

The anthropometric measurements were taken after overnight fasting for a minimum of 12 h (body weight and height analyses with accuracy to 0.1 kg (certified weigh) and to 0.1 cm (stadiometer) in light underwear, with no shoes). Waist circumference was measured at the midway between the costal arch and the upper iliac crest and hip circumference at the level of the greater trochanters. Obtained data were further used to calculate a body mass index (BMI), calculated as weight divided by height squared (kg/m^2^), WHR (Waist–Hip Ratio) calculated as waist measurement divided by hip measurement, and ABSI (A Body Shape Index) calculated by dividing waist circumference by its estimate obtained from allometric regression of weight and height. In addition, body composition measurements were assessed using bioelectrical impedance analysis, carried out with a TANITA BC-418 device (Tanita Corp., Tokyo, Japan). It was used to estimate total body adipose tissue, lean body mass, and water content.

#### 2.2.3. Diet Content Analysis

The study populations also completed nutritional interviews (prospective and retrospective studies). The study used the current recording method to evaluate women’s nutrition, which consists of completing the 3-day dietary diary (first day, fourteenth day, and twentieth day of the study). Then the data was incorporated into dietetics software (DIETA 5.0, recommended by the National Centre of Nutritional Education, Warsaw, Poland) for the calculation of the calorific value of the diet, the amount of micronutrients and macronutrients, including carbohydrates, fats, fatty acids, proteins of animal or plant origin, minerals, vitamins, and fiber.

#### 2.2.4. Physical Activity and Quality of Sleep

Physical activity (number of steps taken, distance travelled) and the quality of sleep for 28 days (sleep and wakefulness over 24 h) were analyzed using a specialized device with motion sensors (Beurer Bluetooth^®^ Activity Tracker AS80 with Health Manager Application, Ulm, Germany).

#### 2.2.5. Biochemical Parameters

Blood samples (5 mL) were collected from each study participant after overnight fasting and an all-night rest between 7:00 and 8:00 in the morning. The blood was allowed to clot (at room temperature, 30 min) prior to centrifugation at 350× *g*. Most of the obtained serum samples were subjected to biochemical analyses immediately after collection, whereas another part of the samples, required to assay proteins and other molecules, was appropriately secured and frozen at −80 °C for further biochemical analyses. Biochemical tests included measurements of glycemia and lipid profile. Fasting blood glucose (FBG) and lipid profile—total cholesterol (TC), high-density lipoprotein (HDL), and triglyceride (TG) levels were determined using enzymatic methods with standardized commercial tests (Cobas c, Roche Diagnostic, Mannheim, Germany). Low-density lipoprotein (LDL) concentration (mmol/L) was calculated as LDL = TC − (HDL + TG/2.2) because triglyceridemia was lower than 4.52 mmol/L [41].

#### 2.2.6. CLOCK and CRY1 Measurement

The analysis of the CLOCK and CRY1 protein concentrations in serum was carried out closely according to the manufacturer’s guidelines, using commercial immunoenzymatic tests (Cloud-Clone Corp., Katy, TX, USA). Microtiter plates with the fixed primary antibody (specific for the determined molecules) were incubated with serum (containing the antigen, namely the measured protein) and later with a secondary antibody marked with peroxidase. Subsequently, a reaction was provoked with a substrate for chromogen, and the color was monitored on an ELISA MR−96 microplate reader manufactured by Clindiag Systems B.V.B.A. (Pollare, Belgium). Protein levels were calculated based on calibration curves which were determined using a 4-parameter-algorithm (SigmaPlot 11.00 software, Systat Software, San Jose, CA, USA) prepared each time for a specific set of assays. Intra-assay and inter-assay coefficients of variation (CVs) were respectively for CRY1 7.1% and 9.2%, for CLOCK 6.2% and 10.0%.

#### 2.2.7. TAS Measurement

The serum parameter of extracellular protection against pro-oxidant activity—TAS (Total Antioxidant Status) was determined by Randox Total Antioxidant Status kits (Crumlin, UK). TAS analysis was based on the incubation of 2.2-azino-di-(3-ethylbenzthiazoline sulfonate) (ABTS) with peroxidase (metmyoglobin) and hydrogen peroxide, which produced a radical cation ABTS+ with characteristic color (changes in absorbance were determined at 600 nm wavelength).

### 2.3. Statistical Analysis

Statistical analysis of the results was conducted using Statistica 13 software with Medical Set (StatSoft, Tulsa, OK, USA). The results are presented as mean ± standard deviations (SDs). The results were analyzed statistically, using elements of descriptive statistics and statistical procedures, such as correlation analysis (with Pearson test for normal and Spearman test for non-normal distributions). Comparisons between groups were performed using a Student’s *t*-test or a Mann–Whitney U test (for respectively normal or non-normal data distributions). Fisher’s exact test was applied for the analysis of the distribution of the qualitative data. Stepwise multiple regression analyses were conducted to determine the predictor for protein levels in blood. The receiver operator characteristics (ROC) curve with area under the curve (AUC) were used to compare the usefulness of different parameters to describe of risk of a small level of CRY1 and CLOCK levels in blood. The normality of the data distribution was assessed using the Shapiro–Wilk test. The level of statistical significance was taken as *p* < 0.05.

## 3. Results

A total of 86 women aged 42.30 ± 9.62 years were enrolled in the study. They were divided into two groups that are comparable in age. The mean BMI of the entire study population was 28.48 ± 6.82 kg/m^2^ and was significantly higher in the HT, at 31.18 ± 6.78 kg/m^2^, than in NHT (25.18 ± 4.11 kg/m^2^, *p* < 0.0001, Table 1). According to the study assumption, the mean SBP and DBP in the study group were significantly higher than in the control group (Table 1). The mean systolic and diastolic blood pressure of all participants were 123.12 ± 19.29 and 76.46 ± 12.10 mmHg, respectively, which were higher in the hypertensive patients (*p* < 0.0000001). The study also showed the presence of metabolic syndrome in a statistically higher percentage of people in HT (*p* < 0.00001).

We found that HT had statistically higher body weight (26% higher; *p* < 0.00003), BMI (24% higher; *p* < 0.0001), and total body fat mass (22% higher; *p* < 0.0000001) as compared with NHT (Table 1). In addition, the study group had significantly higher levels of FBG (14% higher; *p* < 0.00002) and TG (60% higher; *p* < 0.00005). The concentrations of TC and LDL were slightly higher in HT (5.35 ± 1.24 mmol/L and 3.29 ± 1.32 mmol/L) and NHT (4.95 ± 0.95 mmol/L and 2.95 ± 0.9 mmol/L), without a statistically significant difference (*p* > 0.05). Besides, the concentration of HDL was slightly lower in the study group in comparison to the healthy group (1.54 ± 0.40 mmol/L vs. 1.70 ± 0.44 mmol/L), but the difference was not statistically significant (*p* > 0.05) (Table 1).

As shown in Table 1, statistically significant differences between the groups occurred in CRY1 concentration. The blood concentration of CRY1 in HT was 37.38% lower (*p* < 0.01) than in NHT (Table 1). However, no differences were noted in serum CLOCK concentration and TAS status between hypertensive patients and control subjects.

The research carried out showed that patient with hypertension declared similar (to people without this disease) average daily caloric intake and the level of the total amount of carbohydrates consumed daily (1622.07 ± 513.22 kcal/day, 210.78 ± 102.99 kcal/day, 1628.05 ± 384.49 kcal/day, and 215.21 ± 58.32 g/day, respectively, *p* < 0.05), despite larger body weight and BMI. Moreover, the study showed no statistically significant difference between the HT and NHT in the amount of total fat intake (60.69 ± 17.05 and 63.66 ± 19.59 g/day, respectively, *p* > 0.05) or daily protein intake (65.64 ± 16.89 and 67.67 ± 14.93 g/day, respectively, *p* > 0.05).

The study showed that HT was characterized by statistically significant less physical activity during the 24 h than NHT. The average number of steps performed during the day in the study group was 7071.51 ± 1958.11 steps (about 5.37 ± 1.83 kilometers) and in the control subjects 8475.52 ± 3103.42 steps (about 5.79 ± 2.12 kilometers) (*p* < 0.03). Moreover, the study showed no statistically significant difference in the amount of sleep during the day between the studied populations (490.49 ± 105.19 vs. 450.15 ± 92.66 min/day, *p* > 0.05, respectively).

In the entire study population, no relationship was observed between the concentration of CRY1 or CLOCK and biochemical, anthropometric, and lifestyle parameters. The hypertensive patients showed no relationship between the concentrations of CRY1 or CLOCK and biochemical parameters. Of the anthropometric parameters, the concentration of CRY1 was significantly negatively correlated with Fat-Free Mass Index: (R = −0.45, *p* < 0.041) and was significantly positively correlated with the A Body Shape Index (ABSI) (R = 0.40, *p* < 0.035) (Table 2). In addition, a positive correlation was found between CRY1 level and age in this group (R = 0.38, *p* < 0.042). A negative correlation existed between CLOCK concentration and the amount of saturated fatty acids consumed during the day (R = −0.45, *p* < 0.04) in HT. We did not see any association between CRY1 or CLOCK concentrations and the amount of physical activity or sleep quality in the study group. Furthermore, a positive correlation was noted between values of CLOCK and TAS in hypertensive patients (R = 0.46, *p* < 0.036), and between concentrations of CRY1 and TAS (R = 0.47, *p* < 0.032). Similar positive correlations were observed between CLOCK and CRY1 concentration (R = 0.82, *p* < 0.000005) in the HT group.

**Figure 1:**
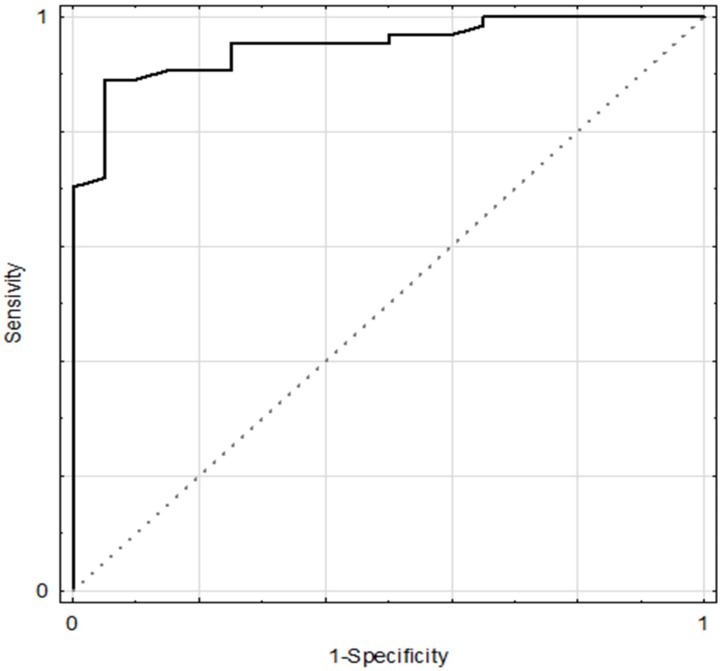
Receiver Operating Characteristic (ROC) curves for CLOCK level for the differentiation between one quarter (Q25) of CRY1 level and the rest group (sum of two, three, and four quarter CRY1 level subgroups) in the HT group—hypertensive individuals.

The study also showed that CLOCK concentration in HT could help to distinguish a risk of one quarter (Q25) with CRY1 level with fair accuracy, as indicated by AUC (Cut-off value < 1.633 ng/mL, sensitivity 89%, specificity 92%, AUC 0.950 ± 0.023, 95%CI: 0.905–0.994, *p* = 0.0001, Figure 1). There was no such relationship in the control group and other parameters.

Moreover, tree variables: SBP, TAS level, and CLOCK level could account for about 18% of variation of CRY1 levels in the entire examined population (HT + NHT) (*p* < 0.0007, R^2^_adj_ (adjusted R-squared) = 18.2%) (Table 3). Analogical relationships did not receive in the case of variation of CLOCK levels.

In NHT in turn, the concentration of CRY1 correlated positively only with body weight and BMI (R = 0.30, *p* < 0.026 and R = 0.35, *p* < 0.009, respectively).

## 4. Discussion

Recent years have shown that circadian mechanisms play an essential role in the regulation of blood pressure. Most of the results regarding the meaning of the circadian clock in BP variability were conducted on rodents, not humans [19,20,23,31,36]. Cry1 gene has been shown to be the primary circadian repressor [42]. Cry1/2 null mice characteristic salt-sensitive hypertension due to the dramatically high synthesis of the mineralocorticoid aldosterone by the adrenal gland [43]. Moreover, a later study demonstrated that Cry1/2 null mice had increased kidney damage on normal and high salt diets [44]. Furthermore, CRY1 interacts with several nuclear receptors and modulate transcriptional activity, suggesting a widespread mechanism of circadian nuclear receptors in the regulation of carbohydrate metabolism [45,46]. A recent study has shown that CLOCK Δ19 mutant mice with partially restored liver clock function under the high-fat diet normalized body weight, energy expenditure, and rescued 24-h food intake rhythms similar to wild-type mice [47]. CLOCK-deficient mice also exhibit reduced arterial blood pressure and altered urinary sodium excretion compared to wild-type mice [48] and have decreased kidney microsomes and urinary levels of 20-hydroxyeicosatetraenoic acid (regulator of BP and blood flow) [49]. Furthermore, CLOCK Δ19 knockout mice have a blunted daily difference in mean blood pressure and a higher prevalence of cardiac hypertrophy and fibrosis compared to wild-type [50]. These studies suggest the role of CLOCK in renal and cardiovascular function. Moreover, Anea et al. demonstrated that a mutation in the CLOCK gene induced endothelial dysfunction was associated with the attenuation of Akt signaling and a subsequent decrease in nitric oxide production that may be significant in the progression of vascular diseases [51]. Data on the concentration of circadian clock protein are scarce [52]. In our study, CRY1 levels in the hypertensive group were significantly lower than in subjects with normal blood pressure. Such a relationship has not been demonstrated for CLOCK concentration. The lack of differences in CLOCK levels in our groups is probably because women with diabetes were eliminated from our study, and abnormalities of this molecule are associated with hyperglycemia and inappropriate blood insulin concentrations (as shown in animal studies [49]). Kovanen et al. indicated that several Cry1 SNPs were associated with metabolic syndrome, arterial hypertension, and elevated blood pressure in humans [53]. Only one more study (besides our research) has evaluated the concentration of CRY1 in the human serum (in two patient groups with obstructive sleep apnea (OSA): with and without primary aldosteronism, PA) and differed significantly from our results (where women with primary aldosteronism or other forms of secondary hypertension were rejected). Tedjasukmana et al. received compared results of CRY1 level in both analyzed groups. Their research has been conducted on a small population: 13 males (M) and three females (F) with OSA + PA and 24 persons (with OSA, without PA; control group: 13 M/11 F) [52].

In our study, CRY1 level was only positively correlated with TAS in the hypertension group (which characteristics high value in such anthropometric parameters as BMI, WHR, and body mass). Some analogs of this relationship may be found in the interesting conclusion of the study of Tedjasukmana et al. that the concentration of this protein in blood decreased in severe hypoxia in OSA patients [52]. Because the destabilization of CRY1 is done by phosphorylation catalyzed by kinase activated by AMP (AMPK) and on the other hand, the activity of this kinase is regulated by the availability of glucose or the inflow of Ca2+ ions into the cell [54,55], so our study group with hypertension and high FGB levels at the same time—may be characterized by low CRY1 concentration in blood. Moreover, CRY1 status (similarly to blood pressure status [56]) in men and women is connected with different mechanisms, which are not fully explained, so further investigation is warranted. 

Of the three parameters analyzed in the multivariable model—SBP level, TAS status, and CLOCK concentration—the latest parameter had the greatest importance in shaping the CRY1 level and proved to be an independent predictor in the entire examined population. Partly these data were also confirmed by the results from the ROC curve for the hypertensive group: low blood concentrations (first quartile, 25%) for CRY1 occurred mainly in subjects with CLOCK concentrations in blood below 1.633 ng/mL (with high values of sensitivity and specificity). This data may suggest a direct relationship between the concentrations of these proteins in the blood, as there is an interaction at their transcriptional level (i.e., CLOCK-BMAL1 complex activates the expression of the cryptochromes gene families during the day) [13,16].

Hypertension is a modifiable risk factor for cardiovascular disease. It often occurs with other cardiovascular risk factors such as central obesity, dyslipidemia, and glucose intolerance [24,40]. The mean BMI in both studied populations was above 24.9 kg/m^2^, which indicates overweight; however, BMI values were significantly higher in women with hypertension compared to the control group. Moreover, HT had a higher body weight compared with NHT. Our findings are supported by previous studies that have identified a direct relationship between BMI and BP that is continuous and almost linear [38]. Some other studies also indicate that BMI showed a significant association with hypertension [57,58,59].

In our study, HT was also characterized by a more frequent occurrence of metabolic syndrome compared to NHT. These results are consistent with the findings of other researchers, as abnormal blood pressure values are a component of metabolic syndrome [60,61]. In the Korean National Health and Nutrition Examination Survey (KNHANES) study, MetS prevalence in patients with hypertension reached almost 60%. Moreover, the presence of metabolic syndrome in the hypertensive population was associated with increased organ damage [61].

The current findings revealed that patients with hypertension had higher values of FBG and TG compared to those without, but TC, LDL, and HDL levels did not differ between the two populations. Different results were obtained in the cross-sectional study with 4202 participants. All lipid profile variables (despite HDL level) and fasting blood glucose level was significantly higher in hypertensive individuals compared to the control group with normal BP [57]. Another study, conducted among 234 participants, showed that serum levels of TC, TG, and LDL were statistically higher, while HDL level was lower in patients with hypertension compared to normotensive subjects [58]. In our study, the insignificantly higher TC and LDL and lower HDL levels in the HT group were most likely due to treatment with lipid-lowering medications (including statins). Therefore there were no statistical differences in cholesterolemia status between the study and control groups.

The present study documented an equivalent value of the concentration of TAS in HT and NHT. The results of other authors’ researches are very diverse. Kharroubi et al. documented increased TAS levels in obese compared to non-obese subjects (2.12 vs. 1.85 mmol/L Trolox). Moreover, this study showed that people with SBP above 140 mmHg had higher TAS levels compared to those with normal SBP (2.15 vs. 1.97 mmol/L Trolox), as in the case with abnormal DBP and the normal DBP values (2.22 and 1.96 mmol/L Trolox, respectively) [62]. Different results were obtained in the study by Chaudharya et al. where they found that TAS levels in hypertensive subjects were significantly reduced (1.74 mmol/L Trolox Equivalent) compared to healthy subjects ≥60 and <60 years old (2.03 and 2.29, mmol/L Trolox Equivalent, respectively) [63]. Antioxidant status is connected with different factors (i.e., age, anthropometric parameters, antioxidative vitamins, and other substances in the diet, and physical activity) [62,64,65]. Because obesity and intensive physical activity promote oxidative stress [62,63,64,65] and our control group was thinner and more active than HT, TAS levels in both groups may be comparable. In the development and treatment of hypertension, the most important modifiable risk factors are diet and physical activity [66,67].

Our study documented lower activity during the day in the HT group than the NHT group, which was statistically significant. Several studies showed that decreased daily physical activity level is associated with a higher risk of hypertension and cardiovascular diseases. Moreover, studies demonstrated beneficial effects of exercise on blood pressure causing reductions in systolic and diastolic blood pressure about 5–7 mmHg [67,68,69].

In our study, the HT and NHT groups declared a similar intake of calories during the day, but the difference between physical activity (22.7% lower value than the NHT group in the number of steps performed during 24 h) suggests a higher energy balance. Studies indicate that restriction caloric intake during the day can lower SBP, DBP, and mean blood pressure compared to a standard diet [70]. This caloric reduction may be a critical change in the studied patients’ lifestyle and could have a measurable impact on blood pressure values. Moreover, the results from a meta-analysis of 17 randomized controlled trials suggest that healthy dietary patterns, such as DASH diet (Dietary Approaches to Stop Hypertension) or a Mediterranean diet, could significantly reduce SBP and DBP (by 4.26 mm Hg and 2.38 mm Hg, respectively). These diets are based on vegetables, fruits, whole grains, legumes, nuts, seeds, fish, and avoiding meat and sweets [71]. Therefore, the diet of the study population should be based on these principles. Moreover, our study has shown a negative correlation between CLOCK concentration and the amount of saturated fatty acids consumed during the day in HT. Animal studies revealed that a high-fat diet alters the function of the circadian clock. [72,73,74]. For example, the consumption of a high-fat diet in CLOCK mutant mice disturbed the circadian rhythm and caused weight gain [72].

In both studied populations of individuals with HT and without, the average length of sleep was around 7.5–8 h per day. A recent meta-analysis has found an association between short sleep duration and hypertension risk. Both excessively longer and shorter sleep periods can be risk factors for this disease, and these relationships are stronger in women than in men. The lowest risk of increased blood pressure occurs with seven hours of sleep a day [32]. These results are confirmed by a later analysis carried out by Grandner et al. with 700,000 adults [75]. Moreover, in a recent cross-sectional study from 2019 involving 19,407 participants, an association was found between short sleep times (<7 h a day) and an increased risk of hypertension [76].

Currently, no data are available on the correlation between CLOCK or CRY1 and anthropometric and biochemical parameters or lifestyle elements to compare our results. Our study is the first analysis of these associations in humans. The study showed a relationship between the concentration of CRY1 and anthropometric parameters in our entire study population. There was also a negative correlation between the CLOCK concentration and the amount of saturated fatty acids consumed during the day in the HT group. Moreover, in hypertensive patients, increased CLOCK and CRY1 values were associated with high TAS levels. On the other hand, we have not shown any association between CLOCK levels in the blood and anthropometric parameters and between CRY1 or CLOCK concentrations and the amount of physical activity or sleep quality.

### Limitations and Strength of the Study

This study had some limitations. The first limitation was the small size of the study and control groups. It is worth considering expanding biochemical analyses to larger research groups (also contain people with diabetes). Moreover, the study contained only female Caucasians, so the results of this study should not be generalized to the general population. Besides, more studies should be done, including more diverse populations (different races, male patients, etc.) to look into the role of circadian clock proteins, especially CRY1, in the regulation of blood pressure. Another limitation is the lack of 24-h blood pressure monitoring.

The key strengths of the study included the in-depth, comprehensive analysis of biochemical, anthropometric, and lifestyle parameters in hypertensive patients. Another strength of this study is the exclusion of patients with secondary obesity or secondary hypertension and obtaining homogeneity of the research group. Furthermore, this is the first study to identify a serum level of CRY1 and CLOCK in hypertensive individuals and its association with anthropometric, biochemical, and lifestyle parameters.

## 5. Conclusions

Our results indicate that hypertensive patients show a decreased serum level of CRY1. However, CLOCK concentration did not differ between NHT and HT populations. Patients with hypertension showed a reduced serum CRY1 level concomitantly to higher body weight, total body fat mass, BMI, FBG, and TG compared to healthy peers. The present study demonstrated the relationship between the concentration of CRY1 and some anthropometric indicators. There was a negative correlation between the CLOCK concentration and the amount of saturated fatty acids consumed during the day in HT, which indicates the possible influence of the diet on the concentration of CLOCK in this population. Furthermore, in hypertensive patients, increased CLOCK or CRY1 values were associated with high TAS levels, which could exhibit their protective effects in these patients. CLOCK concentration in blood had the most significant importance in shaping the CRY1 level and proved to be an independent predictor of CRY1 level in the entire examined population. Further research with a larger study population is required to assess the potential relevance of serum CRY1 and CLOCK levels and the effect on blood pressure regulation. In conclusion, the serum level of CRY1 could be considered in detailed diagnostic of hypertension (primarily in populations with abnormal anthropometric indicators) to help broaden knowledge about arterial blood pressure disorders.

## Figures and Tables

**Table 1:** Anthropometric and biochemical characteristics of hypertensive individuals (HT) and non-hypertensive subjects (NHT).

Parameter (Unit)	HT (*n* = 31)	NHT (*n* = 55)	*p*-Value
SBP (mmHg)	144.10 ± 19.18	114.80 ± 12.13	*p* < 0.0000001 **
DBP (mmHg)	89.81 ± 11.36	71.17 ± 7.8	*p* < 0.0000001 **
Body weight (kg)	90.94 ± 21.65	72.35 ± 13.93	*p* < 0.00003 **
BMI (kg/m^2^)	31.18 ± 6.95	25.19 ± 4.75	*p* < 0.0001 **
WHR	0.88 ± 0.07	0.79 ± 0.06	*p* < 0.0000001 **
Total body fat mass (%)	38.25 ± 6.50	31.38 ± 6.76	*p* < 0.0004 **
FBG (mmol/L)	5.71 ± 1.06	5.03 ± 0.43	*p* < 0.00002 **
TC (mmol/L)	5.35 ± 1.24	4.95 ± 0.95	NS *
TG (mmol/L)	1.58 ± 0.81	0.99 ± 0.57	*p* < 0.00005 **
HDL (mmol/L)	1.54 ± 0.4	1.7 ± 0.44	NS *
LDL (mmol/L)	3.29 ± 1.32	2.95 ± 0.9	NS *
CLOCK (ng/mL)	1.89 ± 0.67	1.96 ± 0.63	NS **
CRY1 (ng/mL)	0.67 ± 0.32	1.07 ± 0.89	*p* < 0.01 *
TAS (mmol/L)	1.32 ± 0.28	1.34 ± 0.25	NS *

Parameters are shown as means (±standard deviations); n—number of individuals studied; p—level of statistical significance for HT vs. NHT groups according to Student’s *t*-test * or Mann–Whitney U test ** (for respectively normal or non-normal data distributions); NS—difference not statistically significant; SBP—systolic blood pressure; DBP—diastolic blood pressure; BMI—body mass index; WHR—Waist–Hip Ratio; FBG—fasting blood glucose; TC—total cholesterol; TG—triglycerides; HDL—high-density lipoprotein; LDL—low-density lipoprotein; CLOCK—circadian locomotor output cycles kaput; CRY1—cryptochrome 1; TAS—total antioxidant status.

**Table 2:** Indices of correlation and levels of statistical significance in cases of analyses the relationship between CRY1, CLOCK, and selected anthropometric parameters in HT.

Parameter (Unit)	Fat-Free Mass Index	A Body Shape Index	TAS (mmol/L)
CRY1 (ng/mL)	R = −0.45 ** p < 0.041 **	R = 0.40 * *p* < 0.035 *	R = 0.47 * *p* < 0.032 *
CLOCK (ng/mL)	NS **	R = 0.49 ** *p* < 0.006 **	R = 0.46 * *p* < 0.036 *

Parameters are shown as R—coefficient of Pearson * or Spearman ** (for respectively normal or non-normal data distributions) and p—level of statistical significance, NS—statistically non-significant difference; HT—hypertensive individuals.

**Table 3:** Comparison of the stepwise multiple regression model explaining variations of CRY1 levels before and after adding the third variable (CLOCK level) to the basic model, which includes SBP and TAS status in the entire examined population (HT + NHT, *n* = 86).

Statistical Parameters	A Model Including Two Variables: SBP and TAS Level	an Extended Model with a New Variable (CLOCK Level)
R^2^_adj_	0.026	0.182
F	1.097	6.415
*p*-value	0.339	<0.0007

TAS—total antioxidant status; SBP—systolic blood pressure; *n*—number of females; R^2^_adj_—adjusted R-squared; p—level of statistical significance in multiple regression; HT– hypertensive individuals and NHT—non-hypertensive subjects.

## Data Availability

The data presented in this study are available on request from the corresponding author. The data are not publicly available due to privacy restrictions.
